# Niche differentiation modulates metabolites abundance and composition in silicon fertilizer amended soil during sugarcane growth

**DOI:** 10.1186/s12870-022-03880-7

**Published:** 2022-10-24

**Authors:** Nyumah Fallah, Ziqin Pang, Fei Dong, Yongmei Zhou, Wenxiong Lin, Kabore Manegdebwaoga Arthur Fabrice, Chaohua Hu, Zhaonian Yuan

**Affiliations:** 1 Key Laboratory of Sugarcane Biology and Genetic Breeding, Ministry of Agriculture, Fujian Agriculture and Forestry University, Fuzhou, 350002 China; 2 College of Agricultural, Fujian Agriculture and Forestry University, Fuzhou, 350002 China; 3 Fujian Provincial Key Laboratory of Agro-Ecological Processing and Safety Monitoring, College of Life Sciences, Fujian Agriculture and Forestry University, Fuzhou, 350002 China; 4 Key Laboratory of Crop Ecology and Molecular Physiology, Fujian Agriculture and Forestry University, Fuzhou, 350002 China; 5 Center for Genomics and Biotechnology, Fujian Agriculture and Forestry University, Fuzhou, China; 6 Province and Ministry Co-Sponsored Collaborative Innovation Center of Sugar Industry, Nanning, 530000 China

**Keywords:** Sugarcane and soil compartments, Metabolites, Silicon fertilizer, Edaphic factors, Sugarcane agronomic traits

## Abstract

**Background:**

As one of the vital crops globally, sugarcane (*Saccharum officinarum* L*.*) has been one of model crops for conducting metabolome research. Although many studies have focused on understanding bioactive components in specific sugarcane tissues, crucial questions have been left unanswered about the response of metabolites to niche differentiation such as different sugarcane tissues (leaf, stem and root), and soil regions (rhizosphere and bulk) under silicon (Si) amended soils. Here, nontargeted metabolite profiling method was leveraged to assess the similarities and differences in the abundance and community composition of metabolites in the different sugarcane and soil compartments. Identify the compartment-specific expression patterns of metabolites, and their association with cane agronomic traits and edaphic factors. We also investigated the response of sugarcane agronomic traits and edaphic factors to Si amended soil.

**Results:**

We found that Si fertilizer exhibited the advantages of overwhelmingly promoting the height and theoretical production of cane, and profoundly increased soil Si content by 24.8 and 27.0%, while soil available potassium (AK) was enhanced by 3.07 and 2.67 folds in the bulk and rhizosphere soils, respectively. It was also noticed that available phosphorus (AP) in the rhizosphere soil tremendously increased by 105.5%. We detected 339 metabolites in 30 samples using LC–MS/MS analyses, 161 of which were classified and annotated, including organooxygen compounds (19.9%), carboxylic acids and derivatives (15.5%), fatty acyls (15.5%), flavonoids (4.4%), phenols (4.4%), and benzene and substituted derivatives (3.7%). In addition, the total percentages covered by these core metabolites in each compartment ranged from 94.0% (bulk soil) to 93.4% (rhizosphere soil), followed by 87.4% (leaf), 81.0% (root) and 80.5% (stem), suggesting that these bioactive compounds may have migrated from the belowground tissues and gradually filtered in various aboveground niches of the plant. We also observed that the variations and enrichment of metabolites abundance and community were compartment-specific. Furthermore, some key bioactive compounds were markedly associated with plant growth parameters and soil edaphic.

**Conclusion:**

Taken together, we hypothesized that Si utilization can exhibit the advantage of enhancing edaphic factors and cane agronomic traits, and variations in metabolites community are tissue-specific.

**Supplementary Information:**

The online version contains supplementary material available at 10.1186/s12870-022-03880-7.

## Background

Sugarcane (*Saccharum officinarum* L*.*) is a major commercial crop with enormous potential. It is widely known as the most important energy and sugar crop globally [1]. Cane is mainly cultivated in tropical and subtropical regions with an annual production of about 16 million tons worldwide [1, 2]. Studies have established that sugarcane growth and development are known to be associated with high nutrient absorption, mainly silicon (Si) fertilizer compared to other nutrients, accounting for about 700 kg ha^−1^ of Si annually [3]. This phenomenon has been attributed to the induced resistance to abiotic [4] and biotic stresses [5].

Si is regarded as the second most abundant element after oxygen in the earth’s crust [6], which accounts for approximately 70% of soil mass [7]. Si is also considered a non-essential element for the development and growth of plants [4, 8]. However, studies have established that Si has an obvious advantage of not only alleviating the detrimental consequences of both abiotic [9] and biotic stresses in different plant species [7, 10] but also improving crop yield [11], nutrients uptake [12]. For instance, Savant et al. [13] indicated that sugarcane amended with Si fertilizer induced resistance against Al, Mn and Fe toxicity mitigation, pest and disease resistance, improved P availability, reduced lodging, improved stalk and leaf erectness and freeze resistance. Decades of research have shown that Si also plays a vital role in plants metabolic and physiological processes [4, 14].

Plants produce a structurally and functionally diverse arsenal of metabolites during their development stages to mitigate the negative effect of different abiotic and biotic factors [15]. Metabolites are considered the basis of organism phenotype and can help understand plant biological processes and their mechanisms more effectively and intuitively. In addition, plants bioactive compounds help them adapt to the continually changing environmental conditions [16], and enhance their overall agronomic parameters [17]. In higher plants, studies have revealed that phenylpropanoid metabolic pathway is one of the main secondary metabolic pathways, consisting of many products, such as flavonoids, hydroxycinnamate amides and lignin, which are cardinal to plant growth and development [18]. Significant progress has been made by the sugarcane research community to identify and characterize the function of the bioactive components in sugarcane [19, 20]. For example, Ezz et al. [21] reported that 42 metabolites including nine fatty acids, nine flavonoids and two sterols were detected in sugarcane juice and molasses. In a related study, an average of 1.10 mg of total flavonoids/g plant material was detected in sugarcane fresh leaves [22]. Despite this progress in understanding bioactive components in specific sugarcane tissues, crucial questions have been left unanswered about the response of niche differentiation of metabolites in different compartments of sugarcane (leaf, stem and root), and soil (rhizosphere and bulk) under Si amended soils, especially ZZ6 variety, one of the widely cultivated sugarcane cultivars in China. To address this knowledge gap, non-targeted metabolomics tool was adopted with the objective of: (i) deciphering the similarities and dissimilarities in the abundance and community composition of metabolites in the different sugarcane and soil compartments, (ii) identifying the compartment-specific expression pattern of metabolites, and assessing their association with cane growth parameters and edaphic factors, iii) investigate the response of sugarcane parameters and edaphic factors to Si amended soil.

## Results

### Response of sugarcane parameters to silicon fertilizer

**Fig. 1:**
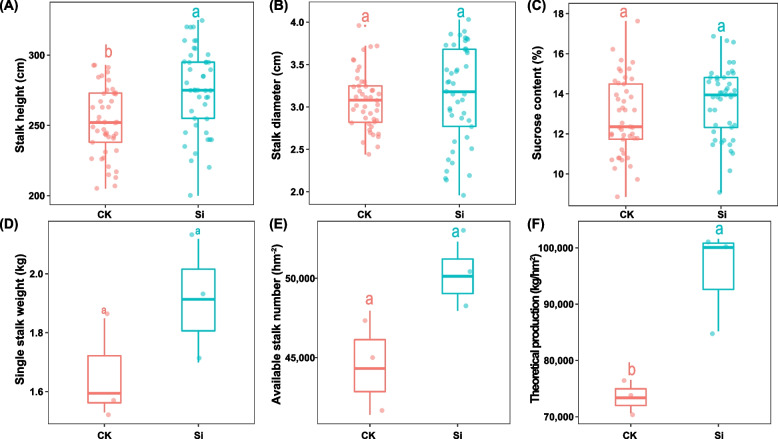
Sugarcane agronomical parameters response to control (CK) and silicon fertilizer (SI); stem height (**a**), stalk diameter (**b**), sucrose content (**c**), stalk weight (**d**), available stalk number (**e**) and theoretical production (**f**)

The application of silicon fertilizer promoted sugarcane growth parameters (Fig. 1). Compared with CK, silicon fertilizer profoundly increased (*p* < 0.05) the height and theoretical production of sugarcane (Fig. 1A, F). The analysis also showed that silicon fertilizer enhanced sugarcane stem diameter, stalk weight and available stalk number relative to those under CK treatment, but showed no significant difference (Fig. 1A, D, E). However, silicon fertilizer had no impact on sucrose content (Fig. 1C).

### Edaphic factors in the different soil compartments response to silicon fertilizer

**Fig. 2:**
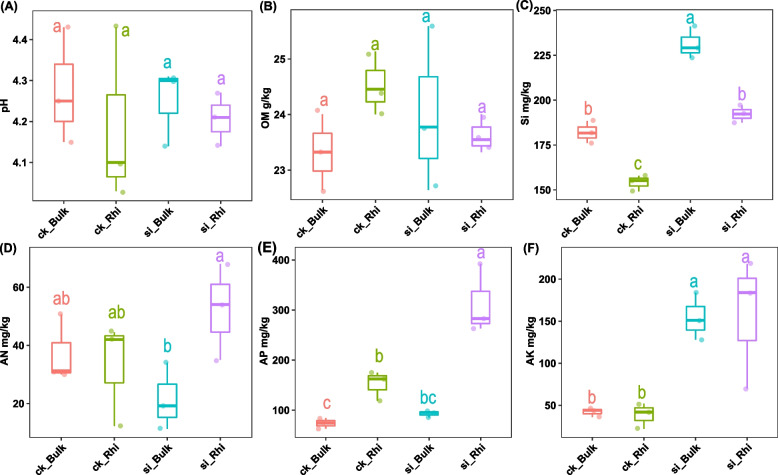
Edaphic factors in sugarcane bulk and rhizosphere soils under silicon fertilizer. Boxes with various lowercase letters signify significant differences between different treatments based on the LSD test (*p* < 0.05). OM, organic matter; SI, silicon; AN, available nitrogen; AP, available phosphorus and AK, available potassium

Edaphic factors under both treatments demonstrated a significant difference in the different soil compartments. Compared to CK, soil Si in the bulk soil significantly increased by 24.8%, followed by 27.0% in the rhizosphere soil (Fig. 2C). Additionally, soil AP in the rhizosphere soil profoundly increased (*p* < 0.05) by 105.5% under Si treatment compared with CK treatment, while soil AN showed little difference in the bulk and rhizosphere soils compared with CK, respectively (Fig. 2D, E). We also noticed that soil AK in both the bulk and rhizosphere soils significantly increased by 3.07 and 2.67 folds under silicon fertilizer treatment compared with CK, respectively (Fig. 2F). However, silicon fertilizer had no significant impact on soil pH and soil OM in comparison with CK treatment (Fig. 2A, B).

### Metabolite abundance and community composition in the different compartments of sugarcane and soil under silicon fertilizer

**Fig. 3:**
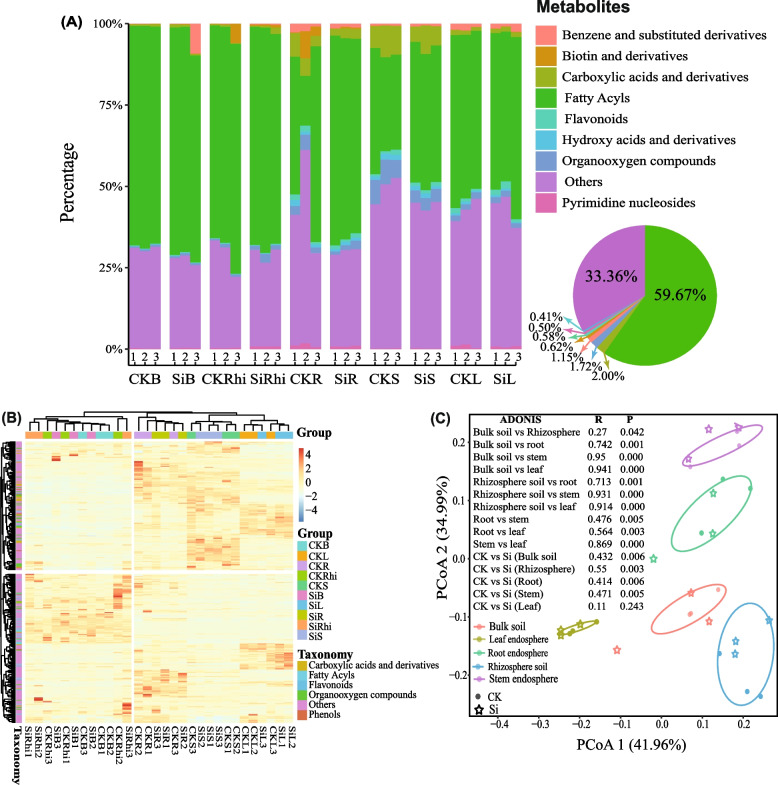
**a** Relative abundance of eight metabolic taxonomy in the entire sample. **b **Principle coordinate analysis (PCoA) with Bray–Curtis distance illustrating similarities or dissimilarities of metabolites community composition in the different compartments of sugarcane and soil under silicon fertilizer (Si) and control (CK), Adonis indicating the significant difference between metabolites in both treatments, and the different compartments (B, bulk soil; L, leaf; R, root; S, stem; Rhi, rhizosphere soil)

Here, we selected eight metabolic taxonomy including fatty acyls (59.67%), followed by carboxylic acids and derivatives (2.00%), organooxygen compounds (1.72%), benzene and substituted derivatives (1.15%), biotin and derivatives (0.62%), flavonoids (0.58%), pyrimidine nucleosides (0.50%) and hydroxy acids and derivatives (0.41%). Metabolites with very low abundance (r > 0.6 and *p* < 0.05) were classified as other (33.36%) (Fig. 3A). Cluster heatmap further showed that carboxylic acids and derivatives, and organooxygen compounds were more expressed both the stem and rhizosphere soil of the sugarcane-intercropped field, whereas flavonoids and fatty acyls were more pronounced in the leaf on the same farming system (Fig. 3B). We then performed multiple difference comparison analysis to test the significant difference of these taxa in the different compartments under both treatments. Compared to CK, it was observed that Si had little or no significant effects on the vast majority of the classified taxa except for organooxygen compounds (*p* < 0.05) (Table S4).

We later constructed cluster heat map to examine the similarity matrix of metabolites in the group of samples. The result showed that the closer the position of the red color, the higher the similarity between samples, while the trend of the green color revealed the opposite. We also observed that the sample correlation within each group was high, with the rhizosphere and bulk soils exhibiting the highest similarity matrix under both CK and Si. Moreover, the average correlation was greater than 0.82, suggesting a good reproducibility of the metabolic composition between samples (Fig. S1, Table S1). We detected 339 metabolites in 30 samples (Table S2), of which 161 were classified and annotated, including organooxygen compounds (19.9%), carboxylic acids and derivatives (15.5%), fatty acyls (15.5%), flavonoids (4.4%), phenols (4.4%), and benzene and substituted derivatives (3.7%) (Table 1). We described the top 20 most abundant metabolites in the various compartments as core metabolites, accounting for 51 in total. The percentages of the total community covered by these core metabolites in each compartment ranged from 94.0% (bulk soil) to 93.4% (rhizosphere soil), followed by 87.4% (leaf), 81.0% (root) and 80.5% (stem) (Table S3).

**Table 1:** Classified metabolites detected in the entire samples

HMDB_taxonomy	Amount	%
Organooxygen compounds	32	19.88%
Carboxylic acids and derivatives	25	15.53%
Fatty acyls	25	15.53%
Flavonoids	7	4.35%
Phenols	7	4.35%
Benzene and substituted derivatives	6	3.73%
Others	59	17.40%

Principle coordination analysis (PCoA) was employed to assess the overall similarity and dissimilarity of the metabolites community composition in the different compartments of the soil and plant tissue under both treatments. The results indicated that PCo1 accounted for 41.96%, while PCo2 represented 34.99% of the total change observed in the metabolites community composition. It was also noticed that metabolites community compositions in the different compartments of soil and sugarcane differed significantly from various treatments. However, metabolites community composition of the leaf under both treatments was more clustered together than in the rest of the other compartments (Fig. 3C).

We conducted two-way ANOVA analysis to have a better understanding of how the various compartments and Si influenced metabolites community. The analysis revealed that the various compartments had a profound impact on the different classes of metabolites. The analysis also demonstrated that the interaction between the different compartments and Si treatment had a considerable impact on the vast majority of metabolites community than Si fertilization. However, both Si and compartments had no impact on benzene and substituted derivatives and biotin and derivatives (Table 2).

**Table 2:** Two-way ANOVA showing the effects of compartment and Si fertilizer on metabolites community

Factors	Fatty acyls	Carboxylic acids and derivatives	Organooxygen compounds	Benzene and substituted derivatives	Biotin and derivatives	Flavonoids	Pyrimidine nucleosides	Hydroxyacids and derivatives	Others
Compartment	***	***	***	NS	NS	***	***	***	***
Si	NS	NS	NS	NS	NS	NS	NS	NS	NS
R*S	***	***	***	NS	NS	***	**	***	***

NS indicates not significant difference

R*S represents the interaction between the different regions and Si treatment. Aesthetic marks, “***” and “**” show significant difference, *p* < 0.05 and *p* < 0.01, respectively

### Differential abundance of metabolites in the various cane and soil compartments under both treatments

**Fig. 4:**
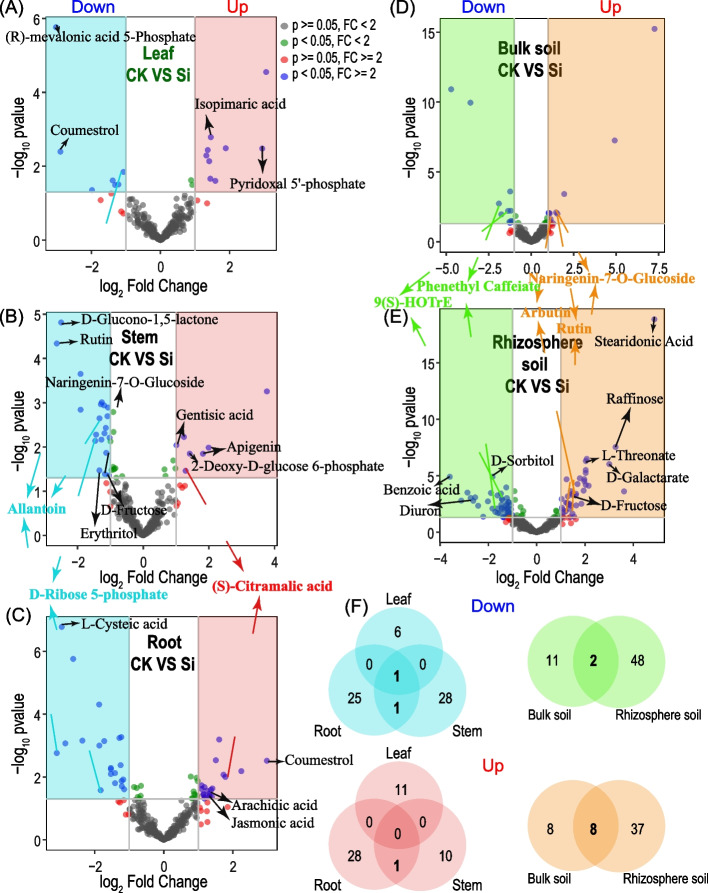
Volcano plots depicting depleted (blue, green) and enriched (red, orange) metabolites compared in two or three groups (**a-e**), followed by a Venn diagram illustrating unique and overlapping enriched or depleted metabolites in the different groups compared (**f**)

Volcano plot analysis was performed to examine the differential abundance of metabolites in the different compartments under both treatments (leaf, CK VS Si; bulk soil, CK VS Si; stem CK VS Si, and rhizosphere soil, CK VS Si) (Fig. 4, Table S5). The result showed that (R)-mevalonic acid 5-phosphate and coumestrol in the leaf depleted in CK as compared to Si, while isopimaric acid and pyridoxal 5'-phosphate improved in Si as compared to CK (Fig. 4A). Moreover, D-glucono-1,5-lactone, rutin, naringenin-7-O-glucoside, allantoin, D-fructose, erythritol and D-ribose 5-phosphate diminished profoundly, while gentisic acid, apigenin, 2-deoxy-D-glucose 6-phosphate and (S)-citramalic acid were significantly enhanced (*p* < 0.05) in the stem relative to those in the CK treatment (Fig. 4B). In the root, L-cysteic acid, D-ribose 5-phosphate and allantoin reduced profoundly in CK, whereas (S)-citramalic acid, coumestrol, arachidic acid and jasmonic acid (JA) were significantly enhanced (*p* < 0.05) under Si as compared to CK (Fig. 4C). In the bulk soil, phenethyl caffeiate and 9(S)-HOTrE decreased considerably in the CK, whereas naringenin-7-O-glucoside, arbutin and rutin increased significantly (*p* < 0.05) in Si than in CK (Fig. 4D). Phenethyl caffeiate, 9(S)-HOTrE, benzoic acid, diuron and D-sorbitol in the rhizosphere soil diminished considerably, while naringenin-7-O-glucoside, arbutin, rutin, stearidonic acid, raffinose, L-threonate, D-galactarate and D-fructose improved significantly (*p* < 0.05) as compared to CK (Fig. 4E).

Venn diagrams analysis was further adopted to quantify the overlap and unique depleted and enriched metabolites community composition in the various compartments. The analysis revealed that 6 (9.83%), 28 (45.90%) and 25 (40.98%) depleted metabolites community compositions were unique to the leaf, stem and root, followed by 11 (18.03%) and 48 (78.68%) in bulk and rhizosphere soils, respectively. However, it was observed that 11 (22.00%), 10 (20%0.00) and 28 (56.00%) of enriched metabolites community compositions were identified in leaf, stem and root, followed by 8 (14.00%) and 37 (69.81%) in the bulk and rhizosphere soils, respectively (Fig. 4F, Table S5).

### Ternary plot analysis revealing enriched or depleted metabolites community in specific sugarcane and soil compartment

**Fig. 5:**
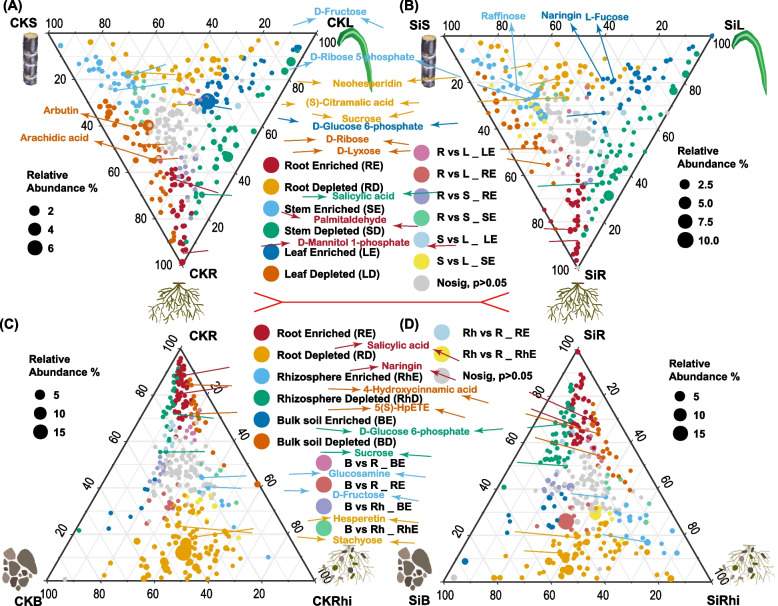
Ternary plot showing enriched and depleted metabolites community in the different compartments. Each point corresponds to the enriched or depleted metabolites. The position of the point denotes its relative abundance in each compartment, and its size represents the average across three compartments. The colored circles represent enriched metabolites in one compartment relative to the other

Ternary plot analysis was then conducted to have a better insight into specific metabolites that were enriched or depleted in various sugarcane and soil compartments by comparing three different compartments, namely, stem, leaf and root under CK (CKS VS CKL VS CKR), and Si treatment (SiS VS SiL VS SiR) (Fig. 5A, B, Table S6). A distinct pattern of metabolites enrichment in the various compartment was observed. Noticeably, D-fructose and D-ribose 5-phosphate showed significant improvement (*p* < 0.05) in the stem of sugarcane under both treatments, whereas raffinose was only enriched (*p* < 0.05) in cane stem under Si compared with the other compartments. On the contrary, salicylic acid in cane stem depleted significantly under both treatments than the other compartments. In sugarcane root, D-mannitol1-phosphate and palmitaldehyde enriched profoundly (*p* < 0.05) under both treatments relative to those in the stem and leaf of the cane. However, neohesperidin, (S)-citramalic acid and sucrose depleted considerably in both treatments. For sugarcane leaf, the analysis demonstrated that D-glucose 6-phosphate enriched significantly (*p* < 0.05) in both treatments compared to the other compartments, whereas naringin and L-fucose were enriched (*p* < 0.05) only under Si treatment than the other compartments. However, D-ribose and D-lyxose diminished under both CK and Si, followed by arbutin and arachidic acid in CK (Fig. 5A, B). Similarly, the enriched or depleted metabolites in the root, bulk soil and rhizosphere soil under CK (CKR VS CKL VS CKRhi), and Si treatment (SiR VS SiL VS SiRhi) were scrutinized (Fig. 5C, D, Table S6). Salicylic acid and naringin in cane root enriched significantly (*p* < 0.05), whereas hesperetin and stachyose reduced profoundly than those in the other compartments under both treatments. In both CK and Si, glucosamine and d-fructose were enriched (*p* < 0.05) in the rhizosphere soil compared to the other compartments, however, D-glucose 6-phosphate and sucrose revealed the opposite. Whereas in the bulk soil 4-hydroxycinnamic acid and 5(S)-HpETE were diminished (Fig. 5C, D).

### Expression pattern of metabolites abundance in the various cane and soil compartments

**Fig. 6:**
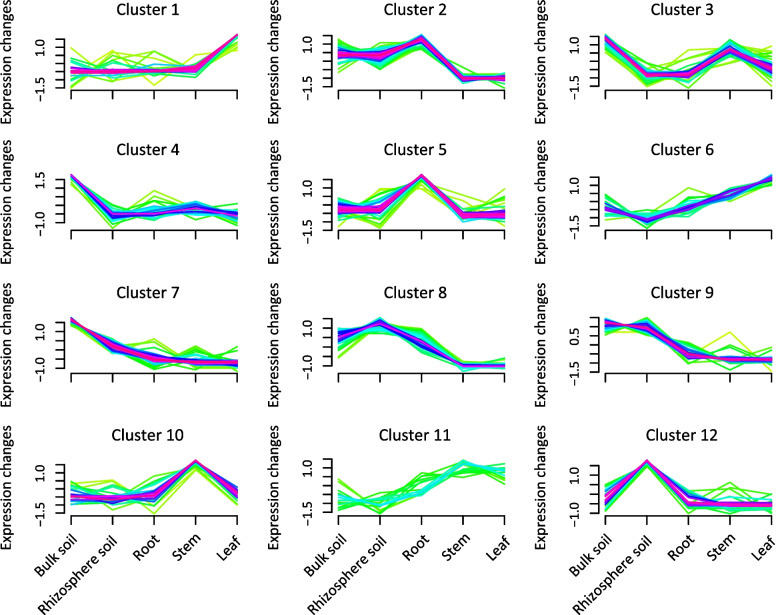
Metabolites abundance expression pattern in various sugarcane and soil compartments. Purple denotes the cluster expression trend of the dominant metabolites, and green signifies low abundance values

In addition, we performed a cluster analysis to decipher the expression pattern of metabolites abundance in the different plant and soil compartments (Fig. 6, Table S7). The results showed that the overall trend of metabolites was divided into 12 clusters. Clusters 1 and 2 revealed higher metabolites abundance in the bulk soil than in other compartments, but cluster 2 peaked significantly (*p* < 0.05) in cane leaf, dominated primarily by cis-9-palmitoleic acid, linoleic acid, palmitic acid, followed by gentisic acid, hypoxanthine, traumatic acid, L-cystine, L-galactono-1,4-lactone and adenine. The metabolites abundance expression pattern peaked in clusters 3 and 10, potentially occupied by D-glucose 6-phosphate, naringin, 3-methoxy-4-hydroxyphenyl-ethyleneglycol, followed by 4-pyridoxic acid, glutathione disulfide, N-carbamoyl-L-aspartate, rutin, uric acid, hydroxyhydroquinone and malonic acid, specifically in the rhizosphere and root, respectively. In clusters 4 and 7, D-ribose, malonic acid, propionic acid, and dihydroxyfumarate, guanosine and N-acetyl-D-glucosamine were enriched only in soil sample. It was also observed that metabolites abundance including D-fructose, D-glucono-1,5-lactone, pyridoxal 5'-phosphate, trans-2-hydroxycinnamic acid, L-aspartate, raffinose, L-glutamine, 2-dehydro-3- deoxy-D-gluconate and L-glutamate expression pattern in cluster 8 and 11 demonstrated an upward trend in cane stem, and declined sharply in the leaf. Moreover, clusters 5 and 6 peak expression patterns of metabolites abundance, namely, cytidine, glucosamine and matairesinol, and arbutin, benzoic acid and L-ribulose showed an upward trend in the root and leaf, respectively. Whereas clusters 9 and 12 demonstrated that metabolites abundance were overwhelmingly expressed in the rhizosphere soil and root, and stem and leaf, potentially driven by 3-isopropylmalate, apigenin and inosine, and pyruvaldehyde and shikimate, respectively.

We performed Pearson's correlation analysis to test the relationship between cane agronomic parameters and metabolites in the various compartments of soil and cane. The result showed that a vast major of the metabolites in specific plant and soil compartments demonstrated a positive association with the various agronomic traits. For instance, in the cane diameter, metabolites such as sucrose and neohesperidin and amentoflavone were positively related to the cane stem and leaf, respectively, while xylitol was positively correlated with both the stem and leaf. Moreover, myristic acid, saccharin and maltitol found in sucrose content were positively associated with the leaf, whereas myristoleic acid showed a positive correlation with both the bulk and rhizosphere soils, while 2-deoxy-D-glucose 6-phosphate exhibited the same pattern with the cane stem. Ammelide, 2'-deoxy-D-ribose and hexadecanedioic acid identified in the yield also revealed a similar trend with the sugarcane stem, leaf and bulk soil, respectively, while rutin and cis-9-palmitoleic showed the same pattern with rhizosphere soil (Table S8).

**Fig. 7:**
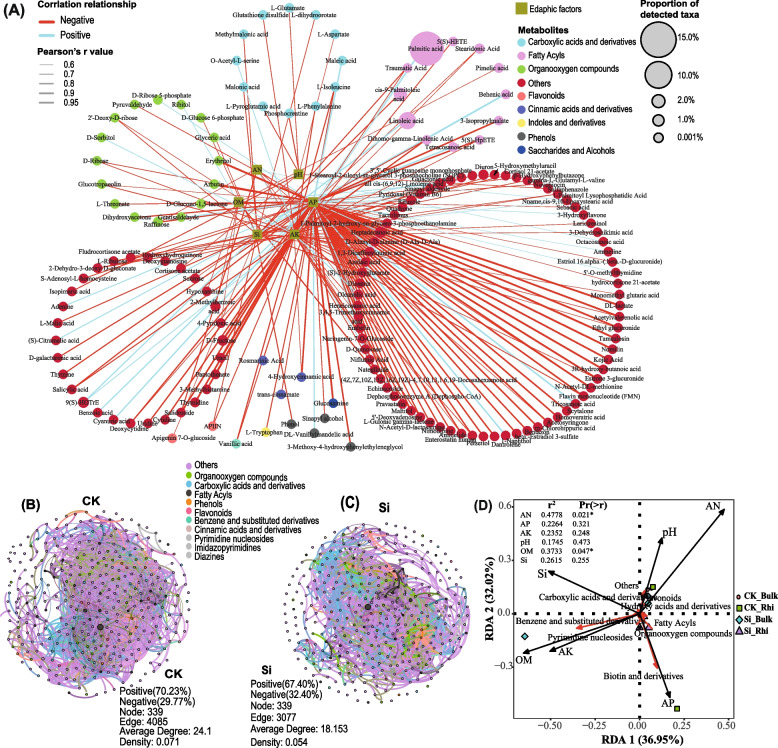
Correlation network analysis depicting the interaction between metabolites and edaphic factors. Red and green lines indicate negative and positive associations, respectively (**a**). Topological features of networks displaying soil–plant associated metabolisms across all sites under CK (**b**), and Si (**c**), based on Spearman correlation method. Redundancy analysis (RDA) shows the association between metabolites and edaphic factors (**d**). Hierarchically clustered heatmap of the all metabolites taxa in the leaf, stem, root, bulk and rhizosphere soils under CK and Si treatments. Variations in the metabolites abundance from the overall mean concentration for each group are shown in red (high abundance) or blue (low abundance) (**e**)

Network analysis was employed to test the relationship between metabolites and edaphic factors. We noticed a vast majority of metabolites were negatively associated with edaphic factors. Specifically, soil AP exhibited a negative association with a substantial portion of metabolites described as other (r < -0.6 and *p* < 0.05), followed by soil AK and Si. Soil AP also showed a negative correlation with a vast majority of the metabolites classified as organoxygen compound, followed by fatty acyles. Similarly, soil pH had a negative association with a vast majority of metabolites classified as carboxylic acids and derivatives. However, soil AP demonstrated a significant and positive association with palmitic acid (Fig. 7A, Table S9). Co-occurrence network was later constructed using random matrix theory (RMT) to measure the dissimilarities in metabolites assemblages in different compartments under both treatments. Dissimilarities were detected between the two networks of metabolites communities under CK and Si. The total nodes and edges in CK (339 and 4085, respectively) were lower than in SR (339 and 3077, respectively) (Fig. 7B, C). The values of all measured modularity indexes in both treatments were larger than 0.4, with Si showing a marked increase, indicating typical module structures. The average clustering coefficient (avgCC) and the average path distance (GD) in CK (0.568 and 3.316, respectively) were higher than Si (0.534 and 3.286, respectively) (Table S10).

RDA analysis further showed that soil AN and pH had a significant and positive impact on hydroxy acids and derivatives, and flavonoids. Furthermore, soil AP significantly and positively influenced biotin and derivatives, whereas OM significantly positively with benzene and substituted derivatives. Soil OM and AN contents were the key environmental factors affecting the composition of soil metabolites. However, Si content demonstrated a negative association with fatty acyls and organooxygen compounds (Fig. 7D). Meanwhile, heat map analysis revealed that the abundance of the top five classified taxa of the various metabolites concentration was compartment-specific (Fig. S1).

## Discussion

Decades of study have largely regarded Si as non-essential nutrients for the growth and development of plants [4, 23]. Contrary to this view, we observed that Si overwhelmingly increased the height and theoretical production of cane, but had no significant impact on the bioactive compounds. This result is in line with findings documented by Meyer and Keeping [24] and Raid et al. [25], wherein it was reported that Si increased cane parameters considerably. Though the mechanism underpinning this phenomenon remains inconclusive, studies have however suggested that it could be ascribed to a series of factors, namely, protection against fungal disease and pests, enhanced water use efficiency [26], high phosphorus absorption, effective and efficient sunlight use through photosynthesis [24]. Previous findings have also validated that Si has the potential to strengthen plant disease and pest resistance by stimulating its natural defense mechanisms induced through metabolites production, such as flavonoid phytoalexins [27].

Si utilization can have a positive outcome on the edaphic factors [28]. Correspondingly, we observed that soil Si content significantly increased by 24.8 and 27.0%, while soil AK was enhanced by 3.07 and 2.67 folds in the bulk and rhizosphere soils, respectively. It was also noticed that AP in the rhizosphere soil profoundly increased by 105.5%. We, therefore, speculated that the active Si may have had a direct positive impact on humification, thereby forming soil organo-mineral compound induced by Si-rich and soil organic matters, which in turn, enhance soil nutrients [29].

Metabolomics is widely regarded as a powerful approach used for identifying, categorizing and examining expression profiles of metabolites in plants [30]. For instance, GC-TOF–MS technique allowed the detection of 73 primary metabolites in a smut susceptible Brazilian commercial sugarcane variety, “RB925345” [31]. In the current study, LC–MS/MS analyses were employed to identify and categorize multiple metabolites in distinct sugarcane and soil compartments. We detected 339 metabolites in 30 samples, 161 of which were classified and annotated, including organooxygen compounds (19.9%), carboxylic acids and derivatives (15.5%), fatty acyls (15.5%), flavonoids (4.4%), phenols (4.4%), and benzene and substituted derivatives (3.7%).

Wijma et al. [32] mentioned that the majority of the dissimilarity identified within the metabolomics profiles was attributed to the differences among the different cane tissues. Here, we noticed a similar trend in metabolites abundance and community composition. The total percentages covered by these core metabolites in each compartment ranged from 94.0% (bulk soil) to 93.4% (rhizosphere soil), followed by 87.4% (leaf), 81.0% (root) and 80.5% (stem). This distribution pattern suggests that these bioactive compounds may have migrated from the belowground compartments and gradually filtered in various aboveground niches of the plant.

We also observed a significantly high abundance of some important metabolites that were unique in some compartments. In the root tissue, we detected a few dominant metabolites; among them was an important plant hormone, JA. JA is an organic compound present in many plants and is widely known as a growth-regulating hormone [33]. According to Creelman and Mullet [34], a significantly high amount of JA levels were identified in the flowers, leaves, and fruit of soybean. This finding suggests that JA may have triggered the proliferation of sugarcane theoretical parameter and height.

In leaf tissue, pyridoxal 5'-phosphate peaked considerably under Si. Pyridoxal 5′-phosphate is considered the cofactor involve in carbanions stabilization at Cα of amino acids, it is also one of the principal dominant factors in amino acid metabolism [35]. In a related study conducted by Hennion [36], pyridoxal 5′-phosphate played a major role in enhancing rice agronomic traits, which we assumed played a similar role in enhancing cane agronomic traits.

In the stem tissue, gentisic acid and apigenin exhibited a significant increase under Si treatment compared with other compartments. Gentisic acid is a metabolite of aspirin, and is widely known to be associated with plant defense. Deseo et al. [37] categorized apigenin as one of the abundant metabolites in sugarcane molasses extract. Apigenin is a flavonoid present in a range of vegetables and herbal spices, namely, chamomile, parsley, basil, cilantro, oregano and celer [38]. It has a multitude of functions, including regulating plant development, as demonstrated in sugarcane growth parameters. This phenomenon further validated that the variations observed in metabolites community and abundance were compartment-specific. Our findings are consistent with previous studies [34, 39], in which they mentioned that the significant differences observed in metabolites community were tissue-specific.

Although ternary plot analysis showed that the vast majority of the enriched and depleted metabolites in the different compartments were common under both treatments, we however noticed that some key metabolites, namely, L-fucose and naringin, and raffinose enrichment in the leaf and stem were unique to the Si treatment, respectively. L-fucose can also activate plants immune system, peptide synthesis, and stomata defense and can control the creation of physical barriers (keratin) [40]. Studies have shown that L-fucose plays a crucial role in the response of plant to abiotic factors such as salt stress, and as a defense mechanism for protein glycosylation. Naringin is one of the important naturally occurring flavonoids, primarily found in plants [41]. It has an antioxidant potential and plays an essential role in the growth and development of many facets of plant physiology, namely, stem, leaves, flowers and buds [42], which was evident in the cane stalk height and theoretical production of the cane amended with Si. Sengupta et al. [43] mentioned that raffinose family oligosaccharides (RFOs) are considered one most significant sets of water-soluble carbohydrates in plant and is well-known to perform desiccation protectant in seeds, storage sugars and transport sugar in phloem sap. Additionally, Li et al. [44] demonstrated that raffinose synthase enhanced plant drought tolerance through raffinose synthesis. These findings conform with the reports documented in previous studies [4, 14], wherein Si had a considerable impact on plants metabolic and physiological processes.

Two-way ANOVA analysis revealed that the various compartments had a profound impact on the metabolites community. Likewise, component-specific abundance and composition of metabolite patterns were also evident in the 12 clusters, where some key metabolites abundance expression patterns in the various compartments exhibited a significant positive correlation with specific plant tissue.

For example, in the leaf tissue, linoleic acid, palmitic acid, followed by gentisic acid, traumatic acid, adenine, arbutin, benzoic acid and L-ribulose were some of the dominant metabolites. For the rhizosphere compartment, D-glucose 6-phosphate, naringin, followed by rutin, uric acid and malonic acid marked a significant increase relative to those in the other compartments. It was also observed that D-fructose, raffinose, D-glucono-1,5-lactone and pyridoxal 5'-phosphate were more prevalent in the stem compared with the other compartments. Whereas D-ribose, malonic acid, propionic acid, guanosine, apigenin and inosine were more pronounced in the bulk soil than those in the other regions, while cytidine, glucosamine and apigenin and inosine peaked significantly in the root tissue. Thus, these results further validate that variations in metabolites abundance and community composition are tissue-specific [39]. We also assessed the association between these metabolites and the different regions using Pearson’s correlations. It was observed that some key metabolites, namely, sucrose, raffinose and palmitic acid demonstrated a positive association with sugarcane diameter, height and stalk number, respectively. We, therefore, postulated that these bioactive compounds could play key roles in plant growth and development [45].

Soil organisms such as plants, use chemical compounds, namely, metabolites to sustain soil fertility and health, which permit them to confront abiotic stress [46]. Rodrıguez-Celma et al. [39] hypothesized that specialized metabolites present in plant roots and exuded to the rhizosphere soil play a key role in the availability of different essential nutrients. Here, we also assessed the relationship between the differential metabolites and soil properties. It was noticed that a vast majority of metabolites were negatively associated with soil properties in the bulk and rhizosphere soils, especially, AP. However, soil AP demonstrated a significant and positive correlation with palmitic acid, which partly agreed with the finding reported by Huang et al. [40], where it was mentioned that nitrogen was markedly associated with flavonoids in plant stalks.

## Conclusion

In summary, our findings demonstrated that Si fertilizer has the potential to s enhance cane agronomic traits and soil edaphic factors, particularly in the bulk and rhizosphere soils. Moreover, the results of this study revealed that metabolites abundance and community compositions varied distinctly in the different soil and plant regions, suggesting that the colonization of metabolites in different plant tissues is compartment-dependent. Furthermore, some key bioactive compounds were significantly associated with plant growth parameters and soil edaphic. Taken together, we postulated that Si utilization can exhibit the advantage of improving edaphic factors and cane agronomic traits, and variations in metabolites community are tissue-specific.

## Materials and methods

### Experimental site, design and treatments

The experiment was conducted in Qumeng Village, Fusui County, Chongzuo, Guangxi Province, China (latitude 22° 49 'N, longitude 107° 76' E). The annual average temperature and rainfall are 21.7℃ and 1121 mm, respectively. Before commencing the experiment, the soil pH was (4.22), organic matter (20.07 g kg^−1^), total nitrogen (1.43 g kg^−1^), total phosphorus (1.25 g kg^−1^) and total potassium (1.18 g kg ^−1^) were assessed on March 12, 2019. The experiment was established in a randomized block design consisting of two treatments with three replicates, which consisted of 6 plots, and a total area of 1800 m^2^ (6 m × 50 m × 6 plots). Sugarcane was cultivated with a line spacing of 1.2 m and 0.1 m row spacing. On March 15, 2019, the soil was plowed (30 cm depth) using rotary tillage, and subsequently followed by cane planting, using the ZZ6 variety, with a seedling rate of about 96,000 / hm^2^. Immediately after the cane was cultivated, the fertilizers were applied. The treatments comprised of: (i) conventional fertilizer (NPK 15–15-15) (CK), applied at the rate of 1500 kg/hm^2^, and (ii) silicon fertilizer (Si), applied at an optimum rate of 1250 kg / hm^2^. The Si fertilizer was a water-soluble compound fertilizer (NPK 18–18-18) consisting of organic silicon and nitrogen, phosphorus and potassium. The organic content of Si was greater than 5%. These soil amendments were produced by Hebei Silicon Valley Academy of Agricultural Sciences.

### Sampling and preparation of root, bulk and rhizosphere soils

**Fig. 8:**
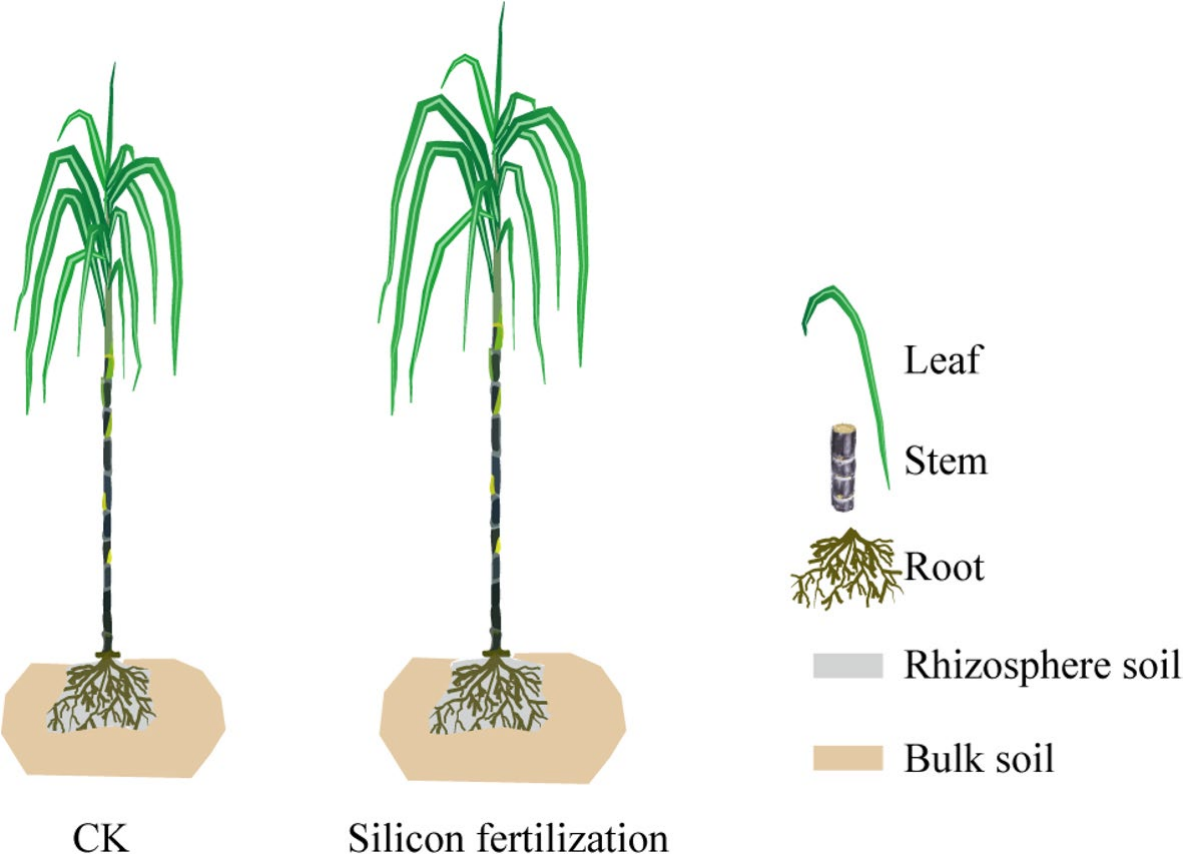
A schematic diagram depicting the different compartments of sugarcane and soil under silicon fertilizer and control (CK)

On 28 November 2019, the bulk and rhizosphere soils, stem, leaf and root (Fig. 8) were sampled using the approach we adopted in previous study [47]. Briefly, soils sampled from the roots of the cane were considered rhizosphere soil. Whereas the bulk soils were sampled 30 cm away from the cane plant roots, with each group of the sample containing three replicates. The samples were then stored in 50 ml Whirl–Pak® bags [48] and subsequently transferred to the laboratory. We then mixed soil of the same replicate, and visible straw, stones and roots were removed. Subsequently, we homogenized the composite samples. A quota of the soil was mixed separately, ground and sieved using 2 mm mesh to test soil environmental variables after air-drying. A random sampling of the biological replicate from each plot was conducted by sampling three healthy cane. The sugarcane roots were collected and washed with phosphate buffer solution. We used 75% alcohol cotton to clean the cane plant leaf and stem, and wrapped in foil, and frozen in liquid nitrogen for 5 min. Lastly, we obtained a total of 30 samples for both the above- and belowground samples.

### Estimation of sugarcane agronomic parameters

We estimated cane heights in centimeters (cm) by using a meter rod from the top of the plant to the soil surface randomly sampling 45 cane in each row. The average of three biological replicates was used to investigate the mean cane height. The sucrose content of cane was estimated following the technique leveraged in Jayanthy study [49]. Whereas each cane stalk weight (kg stalk^−1^) was assessed by measuring the fresh weights of the cane plant. On 28 November 2019, cane plants were harvested, and yield traits were calculated. For the estimation of cane theoretical production, we used the approach adopted in our previous study [47].

### Soil nutrient analysis

Soil environmental variables, including available phosphorus (AP), it was investigated utilizing the Molybdenum Blue approach [50]. We leveraged alkaline hydrolyzable diffusion method to estimate soil available potassium (AK) [51]. Soil pH (1:2.5 soil/water suspensions) was assessed by utilizing Sartorius PB-10 (Germany) [50]. For available nitrogen (AN) estimation, the alkaline hydrolysis diffusion method was utilized [52]. We also evaluated soil organic matter (OM) by employing Walkley–black [53]. Soil Si content was measured by utilizing the approach employed by Babu et al. [54]. In brief, an exact quantity of filtrate was transferred into a plastic centrifuge tube, followed by 10 mL of ddH_2_O, 0.5 mL of 1:1 hydrochloric acid (HCl), and then added 1 mL of 10% ammonium molybdate solution (pH 7.0). We added 1 mL of 20% tartaric acid solution and 1 mL of the reducing agent amino napthol n-sulphonic acid (ANSA), and mixed at 2-min intervals after 5 min. Absorbance was determined at 630 nm by adopting a UV–Vis spectrophotometer (Hach DR 5000) after 5 min, but not more than 30 min after ANSA was added.

### Metabolic samples preparation

Sugarcane samples were prepared following the approach described in previous studies [34, 35]. In summary, a mixer mill (MM400, Retsch) was used to ground the samples into powder for 1.5 min at 30 Hz after they were freeze-dried. Later, we weighed the powder (100 mg) and extraction was done overnight at 4 °C with 0.8 ml 70% aqueous methanol (methanol: H_2_O_2_, 70:30, v/v) and pure methanol, and then centrifuged for 10 min at 10 000 g. The collection of supernatants was carried out separately and mixed, and then filtrated (SCAA-104, 0.22 mm pore size; ANPEL Shanghai, China, www.anpel.com.cn/). To explore the inter-tissue differences in metabolites, samples were mixed into five different tissue samples, namely, leaf, stem, root, bulk and rhizosphere soils for nontargeted metabolomics analysis. To conduct instrument stability, the samples were correspondingly mixed into various quality control samples.

### LC–MS/MS analysis

LC–MS/MS analyses were performed using a UHPLC system (1290, Agilent Technologies) with a UPLC BEH Amide column (1.7 μm 2.1*100 mm, Waters) coupled to TripleTOF 5600 (Q-TOF, AB Sciex). The mobile phase consisted of 25 mM NH_4_OAc and 25 mM NH_4_OH in water(pH = 9.75)(A) and acetonitrile (B) was carried with elution gradient as follows: 0 min, 95% B; 7 min, 65% B; 9 min, 40% B; 9.1 min, 95% B; 12 min, 95% B, which was delivered at 0.5 mL min^−1^. The injection volume was 3μL. The Triple TOF mass spectrometer was used for its ability to acquire MS/MS spectra on an information-dependent basis (IDA) during an LC/MS experiment. In this mode, the acquisition software (Analyst TF 1.7, AB Sciex) continuously evaluates the full scan survey MS data as it collects and triggers the acquisition of MS/MS spectra depending on preselected criteria. In each cycle, 12 precursor ions whose intensity was more than 100 were chosen for fragmentation at collision energy (CE) of 30 V (15 MS/MS events with product ion accumulation time of 50 ms each). ESI source conditions were set as follows: Ion source gas 1 as 60 Psi, Ion source gas 2 as 60 Psi, Curtain gas as 35 Psi, source temperature 650 ℃, Ion Spray Voltage Floating (ISVF) -4000 V in negative modes. The sequencing and library construction were performed by Beijing Biomarker Technologies Co.Ltd. For the bioinformatics analysis, the biomarker biocloud platform (www.biocloud.net) was used in this study.

### Data preprocessing and annotation

MS raw data (.d) files were converted to the mzXML format by adopting ProteoWizard, and processed by R package XCMS (version 3.2). The preprocessing results generated a data matrix that consisted of the retention time (RT), massto-charge ratio (m/z) values, and peak intensity. R package CAMERA was employed for peak annotation after XCMS data processing. In-house MS2 database was adopted for metabolites identification.

### Statistical analysis

Pearson’s correlation coefficients were conducted to examine the association between sugarcane agronomic traits and metabolites. In the various compartments, principle coordinate analysis (PCoA) with Bray–Curtis distance was conducted to test and visualize metabolites community dissimilarities or similarities. To test metabolites dissimilarities between compartments of both treatments, we adopted Permutational Multivariate Analysis of Variance (PERMANOVA) and paired PERMANOVA using “adonis” command in package vegan at 999 permutations and α = 0.05. Volcano plot and ternary plot analyses were employed using the R language-based packages ggtern and grid, an extension of the package ggplot2, to explore enriched metabolites community in the different compartments. Then, we quantified the overlap and unique enriched and depleted metabolites community in the different samples by employing Venn diagrams (http://bioinfogp.cnb.csic.es/tools/venny/index.html). Both R software (http://www.r-project.org/) and Bioconductor (http://www.bioconductor.org/) package ‘Mfuzz’ were used to evaluate metabolites relative expression patterns based on fuzzy c-means. We adjusted the fuzzification parameter to m = 2 and the number of clusters to c = 12 to maintain the soft clustering of all metabolisms. By utilizing a correlation matrix and computing all potential pairwise Spearman's rank using Cytoscape version 3.6.1 [55], we were able to visualize the associations between the abundant metabolites and soil environmental variables in the network. Spearman's correlation coefficient (p) with > 0.7 and the *P*-value was < 0.01 indicated that the correlation between the soil environmental variables and abundant metabolites was significant statistically [56]. We tested the relationship between soil environmental variables and metabolites utilizing redundancy analysis (RDA). We later adopted ‘vegan’ package to test the significance level using 999 permutations [57]. Co-occurrence networks were constructed as described by Zhang et al. [58]. Hierarchical clustering analysis was conducted using R (www.r-project.org/) software with default settings. Z score normalization was adopted to normalize the expression values of metabolites.

## Supplementary Information

**Additional file 1:** **Fig. S1.** Sample correlation within each group of sample.

**Additional file 2:** **Table S1.** Sample correlation within each group of sample. 

**Additional file 3:** **Table S2.** Percentages of metabolite abundance in the 30 samples. 

**Additional file 4:** **Table S3.** Total percentages of metabolite community abundance in each compartment. 

**Additional file 5:** **Table S4.** Comparison of metabolites relative abundance in each group. 

**Additional file 6:** **Table S5.** Changes of metabolic composition in different compartments under Si and CK. 

**Additional file 7:** **Table S6.** Changes in metabolic composition in different compartments.

**Additional file 8:** **Table S7.** Expression pattern of metabolites abundance in the various cane and soil compartments. 

**Additional file 9:** **Table S8.** Pearson’s correlations between different compartments and metabolite composition. 

**Additional file 10:** **Table S9.** Association of metabolites with edaphic factors. 

**Additional file 11:** **Table S10.** Co-occurrence network depicting the dissimilarities of metabolites in the different compartments under both treatments.

## Data Availability

All data generated or analyzed during this study are included in this article and additional information is available from the authors upon request.
