# Supplementary figures and images for: Niche differentiation modulates metabolites abundance and composition in silicon fertilizer amended soil during sugarcane growth
Source: BMC Plant Biol. 2022 Oct 24;22:497. doi: 10.1186/s12870-022-03880-7 (PMC9590199; doi:10.1186/s12870-022-03880-7)

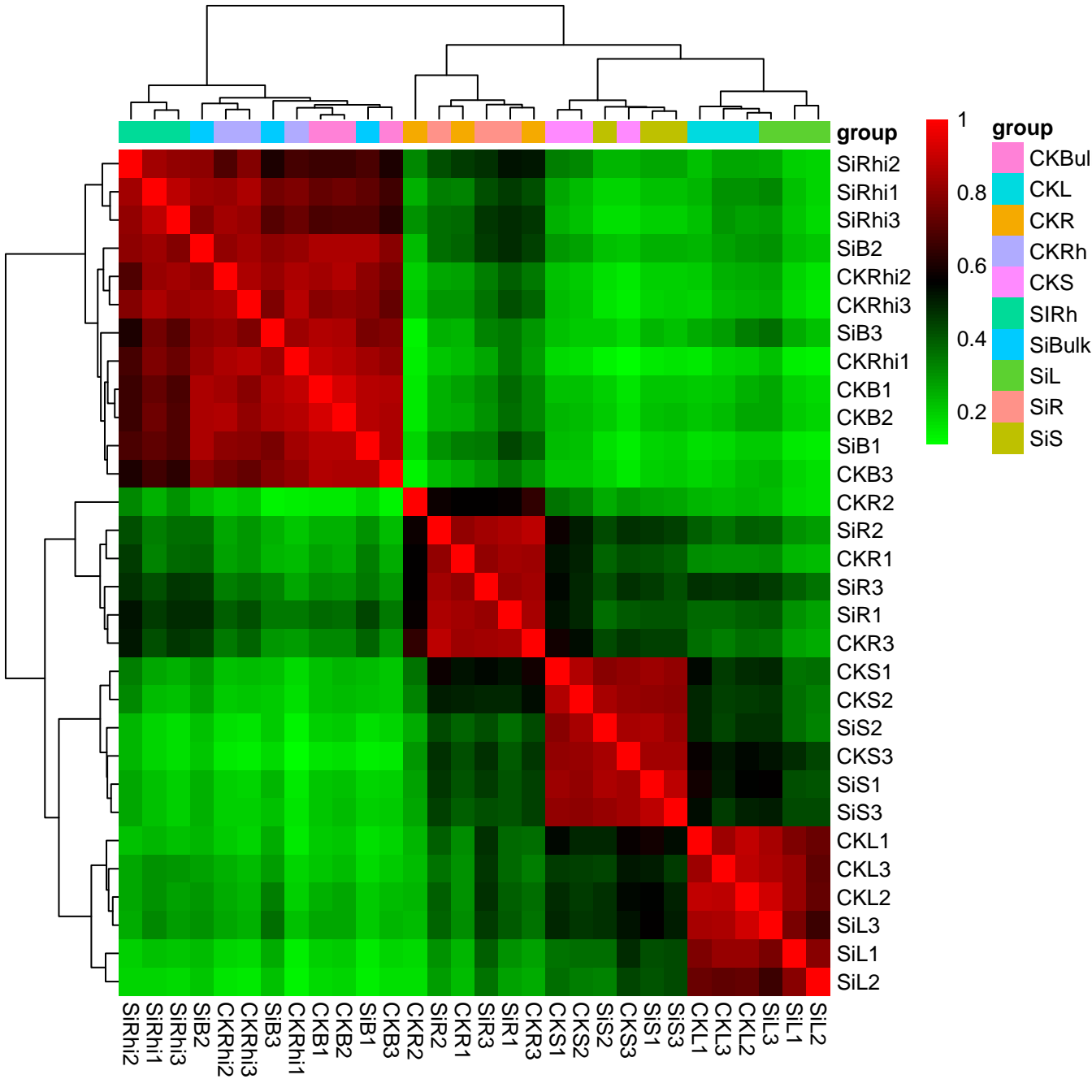

Supplement: Supplementary file 1 — Additional file 1: Fig. S1. Sample correlation within each group of sample. [file 12870_2022_3880_MOESM1_ESM.pdf]
